# Compensation for cures: Why we should pay a premium for participation in ‘challenge studies’

**DOI:** 10.1111/bioe.12596

**Published:** 2019-05-28

**Authors:** Jonathan Anomaly, Julian Savulescu

**Affiliations:** ^1^ Institute for Practical Ethics UC San Diego 9500 Gilman Dr. 92093 La Jolla CA United States; ^2^ Uehiro Centre for Practical Ethics University of Oxford Suite 8 Littlegate House16‐17 St Ebbe's Street OX1 1PT Oxford United Kingdom of Great Britain and Northern Ireland

**Keywords:** antibiotic resistance, antibiotics, challenge studies, consent, phage therapy

## Abstract

Antibiotic resistance is one of the most pressing public health problems humanity faces. Research into new classes of antibiotics and new kinds of treatments – including risky experimental treatments such as phage therapy and vaccines – is an important part of improving our ability to treat infectious diseases. In order to aid this research, we will argue that we should permit researchers to pay people *any* amount of money to compensate for the risks of participating in clinical trials, including ‘challenge studies’ that involve deliberately infecting patients. We think that standard worries about paying for participation in risky research are reducible to concerns that can be addressed with the right screening mechanisms.

## 1 HOW CHALLENGE STUDIES WORK

One of the greatest achievements in medicine is the eradication of smallpox through the smallpox vaccination. During the 20th century alone, smallpox is believed to have killed somewhere between 300 and 540 million people – 3 times more people than were killed by all the wars of that period.1Oldstone, M. (2010). *Viruses, plagues, and history*. New York, NY: Oxford University Press. It has a mortality of 30% and is highly infectious. Those who survive are often left horribly scarred or blind.

The vaccine developed by Edward Jenner brought this tragedy to a halt. In 1796, Jenner infected 8‐year‐old James Phipps with cowpox, a related but far less virulent virus. This was a challenge study. Challenge studies involve deliberately infecting human beings with a microbe in order to study its pathogenesis, or to test the efficacy of a vaccine or an antimicrobial medication. In the case of Phipps, the cowpox conferred an immunity to smallpox. Jenner subsequently infected Phipps with smallpox, but showed he had developed immunity. This provided strong evidence for the idea that infection with cowpox conferred immunity. He called this process vaccination.2Riedel, S. (2005). Edward Jenner and the history of smallpox and vaccination. *Baylor University Medical Center Proceedings, 18*, 21–25. This was the birth of immunology and of vaccination in the modern era, though there is evidence that ancient Chinese, Indians and medieval Europeans developed a primitive vaccination, which consisted of infecting themselves with the dried scabs of people who were dying of smallpox.3Crawford, D. (2009). *Deadly companions: How microbes shaped our history*. Oxford: Oxford University Press.


Challenge studies remain important in facilitating medical advance. For many years, peptic ulcers were thought to be caused by stress. But in 1984 an Australian doctor named Barry Marshall, who hypothesized a causal connection between ulcers and bacteria, infected himself with *Helicobacter pylori*. Within a week, Marshall developed gastritis, which he subsequently cured with antibiotics. He received the Nobel Prize in Medicine in 2006.4Hellstrom, P. (2006). This year's Nobel Prize to gastroenterology. *World Journal of Gastroenterology, 12*, 3126.


Challenge studies offer significant benefits. For example, in the investigation of candidate vaccines, physiological or biochemical signs of infection can be detected before symptoms develop. So it is possible to prioritize the safety of participants, limit the number of research participants, and study the most promising vaccine candidates. Instead of testing vaccine efficacy on thousands of participants, testing can be done with fewer than 100 in hospital settings.5Bambery, B., Selgelid, M., Weijer, C., Savulescu, J., & Pollard, A. (2016). Ethical criteria for human challenge studies in infectious diseases. *Public Health Ethics, 9*, 92–103. Not only can this be safer for participants, it can also involve significant savings of resources. Indeed, because a larger number of potential vaccines can quickly be tested, it also increases the chances of identifying an effective vaccine.

Challenge studies can thus reduce the number of participants exposed to the risks of research. In many diseases, such as malaria, less than 10% of promising vaccines proceed to phase III trials.6Davis, M., Butchart, A., Coleman, M., Singer, D., Wheeler, J., Pok, A., & Freed, G. (2010). The expanding vaccine development pipeline. *Vaccine, 28*, 1353–1356. Any trial of a new intervention has risks associated with the new intervention, and risks associated with being denied effective treatment for a disease by being given a placebo or an ineffective intervention. Challenge studies reduce the numbers of people exposed to such risks.

## 2 CHALLENGE STUDIES AND ANTIMICROBIAL RESISTANCE

Challenge studies can offer a new strategy in the war against antimicrobial resistance. New antibiotics have been difficult to develop in the last few decades, in part because we have already found and deployed many of the most basic biochemical weapons in nature. According to a recent analysis, ‘nearly all antibiotics in use today are compounds that were discovered during the 1940s to 1960s – the golden era of antibiotic discovery – or their derivatives.’7Lewis, K. (2013). Platforms for antibiotic discovery. *Nature Reviews Drug Discovery, 12*, 371–387, p. 372. Most new antibiotics derive from the basic biochemistry of antibiotics discovered decades ago, with the exception of a small number of synthetic compounds such as fluoroquinolones.

In fact, although synthetically created antibiotics have some promise, they will likely have a limited effect in slowing our evolutionary arms race with pathogenic bacteria. The reason is best explained by Brad Spellberg, past president of the Infectious Diseases Society of America:

…after billions of years of evolution, microbes have most likely invented antibiotics against every biochemical target that can be attacked – and, of necessity, developed resistance mechanisms to protect all those biochemical targets. Indeed, widespread antibiotic resistance was recently discovered among bacteria found in underground caves that had been geographically isolated from the surface of the planet for 4 million years. Remarkably, resistance was found even to synthetic antibiotics that did not exist on earth until the 20^th^ century. These results underscore a critical reality: antibiotic resistance already exists, widely disseminated in nature, to drugs we have not yet invented.8Spellberg, B. (2013). The future of antibiotic resistance. *New England Journal of Medicine, 368*, 300.

Because the existing stock of antibiotics is dwindling, and the rate of discovering new antibiotics has slowed, it is likely that scientists will turn to more radical cures such as phage therapy, new kinds of vaccines, immuno‐enhancements, and experimental treatments that are especially risky in their early phases. We will explore the ethics of paying people to participate in risky trials, including challenge studies, that test novel treatments for bacterial infections.

## 3 COMPENSATION FOR CURES

The idea of paying people who agree to be infected with live bacteria, or inoculated microbes, is bound to strike some people as outrageous. The point of medicine, after all, is to treat disease, not induce it. But drugs and vaccines must be tested for safety and for efficacy, and unless we pay a premium to attract participants, the progress of new treatments will be slowed, and the quality of scientific research might be compromised. We think that once some basic moral requirements for recruiting subjects are met, we should let researchers pay any amount to recruit and retain participants in microbial challenge studies.

In an ordinary market, prices emerge through the independent choices of buyers and sellers, and they reflect the value diverse people place on the alternative use of scarce resources. In markets for research subjects, prices are set by Institutional Review Boards (IRBs), but they are sensitive to people's willingness to participate. Willingness to participate is, in turn, partly a function of people's available alternatives and their attitude towards risk.

We tend to respect people's choices to engage in work such as mining coal and building skyscrapers, provided they freely consent to the relevant risks. Society gains the benefits of cheap energy and affordable accommodation, and the workers are paid a price that reflects the dangers they are willing to incur for additional income. Similarly, once appropriate conditions are in place to regulate challenge studies, nearly everyone – especially future people – benefits from research that ultimately pays off by reducing the burden of infectious disease.

Of course, no market is fully free. Labor markets occur in the context of regulations that constrain how workers can be treated by companies, how much pollution a company is allowed to produce, and the conditions under which a worker can sue a company for breach of contract. These background regulations are intended to prevent unjust exploitation of workers, to prevent harm to third parties, and to ensure that markets work for the good of all. The same thing goes for regulations set by IRBs and ethics committees: if they have done their job well, the amount subjects are paid is irrelevant. Indeed, as we will argue, one important danger is that research participants are exploited by *being paid too* *little*.9Savulescu, J. (2001). Taking the plunge. *New Scientist*,* 2280*, 50. Retrieved from https://www.newscientist.com/article/mg16922805-200-taking-the-plunge/. Accessed Jan 1, 2019.


### 3.1 The meaning of money

Many ethicists have registered discomfort about enticing people to do things for money that they think it is permissible – even praiseworthy – to do for free. For example, Elizabeth Anderson suggests that although it is fine to carry an infertile friend's baby to term by acting as a surrogate, *paying* someone to participate in a surrogacy contract is morally dubious.10Anderson, E. (1990). Is women's labor a commodity? *Philosophy and Public Affairs, 19*, 71–92. Similarly, Michael Sandel thinks that although there is nothing wrong with asking friends for advice on how to write wedding vows, paying someone to write them is wrong.11Sandel, M. (2003). What money shouldn't buy. *The Hedgehog Review, 5*, 77–97. These examples have some intuitive plausibility, and our intuitions about cases like this reflect the fact that payment may introduce crass motivations or corrupt either the thing being sold (in the case of a wedding speech) or the nature of a relationship (in the case of surrogacy contracts). But intuitions can be misleading.

In a recent book, Jason Brennan and Peter Jaworksi challenge the view that money introduces any *new* wrongness into an exchange. They argue that, morally speaking, if you can do it for free, you can do it for money.12Brennan, J., & Jaworski, P. (2015). *Markets without limits: Moral virtues and commercial interests*. New York, NY: Routledge Press. In most standard cases of morally troubling markets it is the nature of the exchange – not the fact that money is involved – that troubles us. For example, markets for kidnapping are wrong because it is wrong to abduct children, not because it is done for money.

We can imagine cases in which a market is morally dubious because the items being exchanged are not the kinds of things that should be for sale (like children), cases where the parties to exchange are under duress (as when a thief puts a gun to your head and asks ‘your money or your life?’), or where the initial conditions that generate the exchange are unfair (for example, if a company dumps pollution in your backyard and then asks how much you would pay for them to stop polluting). But in some cases, even when problems like this are absent, some people think that the mere presence of money alters the nature of the exchange in ways that make it morally suspicious. For example, Sandel and Anderson seem to think that buying wedding vows or renting a womb are wrong at least in part because they express disrespect for the sanctity of marriage or the proper relationship between mother and child.

But as Brennan and Jaworksi emphasize, the idea that money ‘expresses’ an attitude, or symbolically alters a relationship, suggests that we are making inferences based on local social norms rather than moral judgments that apply to all societies. For example, in the United States it is considered praiseworthy among some people to pay children to do housework (some children receive a weekly ‘allowance’ for helping their parents do laundry or clean the house). But in some countries, or some subcultures within a country, this would be considered wrong: children should obey their parents not because it is profitable, but because it is right.

Sometimes local norms lead us to think of certain kinds of exchanges as wrong, even when the consequences are good and the relevant parties consent. For example, in some cultures it is considered wrong to pay a nanny to take care of children during the day so that their mother can work. But since nannies allow women independence, that is a defect of local norms, not a good objection to paying for childcare. When local norms are oppressive, that may give us reason to try to change the norms rather than criticize people who disobey them.13Anomaly, J., & Brennan, G. (2014). Social norms, the invisible hand, and the law. *University of Queensland Law Journal, 33*, 263–283.


We argue that once the right background conditions are in place, if you can do it for free, you can do it for *any amount* of money. In particular, once certain ethical criteria are met in recruiting participants for challenge studies, adding large amounts of money to the exchange adds no new wrongness, and may have tremendous benefits, ranging from making participants better off to having a more representative sample to study. We concede that without the right background regulations in place, offering large amounts of money can have objectionable consequences.

### 3.2 Research protocols

There is a fairly strong consensus on moral protocols that should govern clinical research. For any study involving human subjects, many in the medical community agree that IRBs should try to ensure that participants give informed consent, that they are selected according to a fair process, that there is sufficient social value to the experiment, that the study uses appropriate scientific principles, and that the experiment has a favourable risk–benefit ratio so that whatever risks participants are exposed to are offset by benefits to themselves or other people.14Emanuel, E., Wendler, D., & Grady, C. (2008). An ethical framework for biomedical research. In E. Emanuel (Ed.), *The Oxford textbook of clinical research ethics* (pp. 123‐135). Oxford: Oxford University Press. There is disagreement around the edges, but there is widespread consensus on the core principles.

Additional protocols have been proposed for microbial challenge studies, which are potentially more dangerous than standard medical trials, given the nature of infectious disease. Challenge studies are unique because, unless participants are adequately supervised, participants can infect non‐participants.15Miller, F., & Grady, C. (2001). The ethical challenge of infection‐inducing challenge experiments. *Clinical Infectious Diseases*,* 33*, 1028–1033. Thus, in addition to the core principles governing clinical research, some argue that IRBs tasked with licensing studies that involve deliberate human infection should also make sure (a) that there are mechanisms that protect non‐participants; (b) that there is a compensation scheme for participants harmed by infection; and (c) that the relevant studies are transparent so that outside parties can monitor both the methods and the results of the study. This is especially important for maintaining public trust in the methods being used, and in the safety and efficacy of the products that emerge from challenge studies.

For the most part, we endorse these requirements (though we are a little skeptical of the idea that ‘experts’ can establish the right level of risk to expose research subjects to). But we want to challenge the common argument that the amount of payment should be considered an important moral consideration in drawing volunteers to participate in risky research.

### 3.3 Exploitation

One of the commonest objections to paying participants large amounts of money to take part in risky research is that this will exploit the poor. However, this objection goes the wrong way. The poor are exploited when they are paid too little to do valuable work that involves risk, such as working on high‐rise scaffolding or in coal mines. The appropriate response is to ensure minimum safety requirements and to pay a minimum premium for risk: danger money.

If any amount of money is paid, it should reflect the risks that informed adults are willing to take in a market setting under conditions in which information is symmetric. This speaks in favour of setting a fair minimum for compensation for some research participation, especially when information is unreliable or asymmetric. Paying above this is not a problem: it is like paying people more to attract them to other kinds of risky or unpleasant work.

There are two ways to address exploitation. The first is to correct the background injustice that leads the poor or underprivileged to accept risks they would not otherwise take on. The second is to pay them a fair price for their labour. Research involving risk can be seen as a kind of labour and should be compensated appropriately.

### 3.4 Undue inducement

We agree that coercion and exploitation undermine consent, and that desperation by participants or deception by researchers can make consent invalid. But we are skeptical of the idea that money affects consent when appropriate checks are in place. There are a handful of variations on the idea that money can corrupt choices or outcomes.

#### 3.4.1 Cloudy judgments

Most medical ethicists do not think that paying for research is intrinsically wrong. But many worry that offering *too much* money can cloud a potential participant's judgment in ways that weaken consent. For example, Christine Grady argues that ‘large amounts of money designed simply to entice, to outbid other studies, or to make up for risk should not be allowed’ because it can ‘distort judgment and push people toward deception’.16Grady, C. (2001). Money for research participation: Does it jeopardize consent? *The American Journal of Bioethics*,* 1*, 43.


Grady's worries are reasonable, but we think that if prospective participants understand the risks, we do not need experts trying to figure out whether offering ‘too much’ money might undermine voluntary choice. Even if it is possible that in some cases, offering more money would make some people discount risks in a way that they themselves might disavow if they were thinking clearly, we doubt that experts are better equipped in any given case to make this choice for them. We worry that members of IRBs will lack the kind of intimate information people have about themselves, and will be prone to substitute their own judgment for that of the participant. Given the incentive and information problems third parties face, tasking experts with deciding whether a certain amount of money constitutes ‘undue inducement’ may end up being an instrument for paternalism.17Savulescu, J. (2001). The fiction of ‘undue inducement’. *The American Journal of Bioethics, 1*, 1–3.


Consider, for example, what would happen if we ran a study on a new antibiotic that satisfies all of the protocols mentioned above. Now imagine that the treatment has the potential to save many lives, but that it is very risky, which makes it hard to recruit participants. It is difficult to quantify risks in clinical trials. But assume for the sake of argument that the trial involves a 10% chance of death, and that very few people will participate with an offer of free medical treatment for life, or an offer of $10,000 in cash. How would experts know whether offering another $5,000 would make someone less capable of thinking through the relevant trade‐offs?

Apart from thought experiments, some actual experiments (using survey data from hypothetical scenarios) suggest that varying up the amount offered to patients for participation in clinical trials does *not* alter participants’ ability to process information about risk.18Halopern, S., Karlawish, J., Casarett, D., Berlin, J., & Asch, D. (2004). Empirical assessment of whether moderate payments are undue or unjust inducements for participation in clinical trials. *Archives of Internal Medicine, 164*, 801–803. See also Singer, E., & Couper, M. (2008). Do incentives exert undue influence on survey participation? Experimental evidence. *Journal of Empirical Research on Human Research Ethics, 3*, 49–56. While increased payment may alter participation rates by signaling that a procedure is more risky19Cryder, C., London, A., Volpp, K,. & Lowenstein, G. (2010). Informative inducement: Study payment as a signal of risk. *Social Science and Medicine*,* 70*, 455–464. , this is a perfectly rational inference: ordinary people understand that more money is generally required to induce people to incur greater risk. It is possible, of course, that conditions like extreme poverty can alter perceptions of risk for some people. But as argued above, the protocols for challenge studies already attempt to filter out people whose desperation or compromised capacities render them incapable of understanding the relevant risks.

#### 3.4.2 Bad results

Quite apart from worries about large amounts of money undermining consent, it is possible that offering too much money might jeopardize the scientific validity of a study by inducing participants to conceal medical conditions that might be relevant. For example, if I can make a lot of money by participating in a clinical trial for a new antibiotic, I might lie about the fact that I have an immune system compromised by some other infection that might alter the efficacy of the antibiotic.

This is a real problem. But rather than altering the payment, IRBs can instead turn to more objective metrics of a participant's fitness for a trial,20Wilkinson, M., & Moore, A. (1997). Inducement in research. *Bioethics*,* 11*, 373–389. including genetic testing and registries that record which other trials a participant might be enrolled in.21Largent, E., & Lynch, H. (2017). Paying research participants: the outsized influence of “undue influence.” *Hastings Center Report*,* 39*, 1–9. In the kinds of studies we have in mind, IRBs would likely conduct blood tests to detect the presence of other infections that might be relevant, or microbiome examinations to obtain a representative sample of which bacteria a participant is colonized with.

Finally, it is important to recognize that capping the amount of money offered for a trial might shrink the pool of participants to an unrepresentative sample. For example, below some threshold it may be that only very poor people will participate, and they may be more likely to have conditions that they fail to disclose. Putting the point another way, offering something more like the market rate for participation may be more likely to draw a diverse pool of people. This allows IRBs to focus on the scientific merits of the research they are tasked with approving, and with moral protocols that can be applied in a more objective and less paternalistic way.

#### 3.4.3 Local norms

Some bioethicists have taken a different tack, arguing that it is not the amount of money relative to a person's income that might undermine consent, but rather that consent can only be given if it is consistent with local norms that determine appropriate payment. For example, Ezekiel Emanuel *et al*. argue that ‘recruitment procedures and incentives for participants should be consistent with cultural, political, and social practices of the potential participants’ and that ‘the appropriate form and level of compensation depends upon the local economic and social context’.22Emanuel *et al*. *op cit*. note 14, p. 131.


We disagree with this argument.

First, not everyone within a culture agrees with the norms of the group they are born into, nor should they. There are eccentric individuals who do not care much about local norms that dictate whether we can pay for an activity in cash or in kind. There are also conscientious objectors who reject the semiotics of their society. For example, some people in Saudi Arabia think that women should not appear in public without an escort, and others think that women should not be permitted to use birth control or study biology and become physicians. We think that women who disobey these norms should be praised rather than blamed, even if a majority of those around them disagree with their choices.

Second, individuals rather than cultures are the proper object of respect (though we should also recognize that culture has some independent value, and is a product of the individuals who create and sustain it). A medical study exposes *individuals* to risks and rewards, not the various groups with which she identifies. As such, it is the individual, not any of the groups she considers herself a member of, that should elect the proper level and kind of compensation. Of course, no individual can demand any amount of money, but each should be free to respond to offers without other members of her culture, or the norms of a particular culture she happened to be born into, having veto power over her choice.

#### 3.4.4 Money and trust

A final worry about paying large amounts of money to entice people to participate in challenge studies is that trust in the medical research community, and in the vaccines and drugs they produce, might be undermined when participants are exposed to serious risks.23Hope, T., & McMillan, J. (2004). Challenge studies of human volunteers: Ethical issues. *Journal of Medical Ethics*,* 30*, 110–116. The worry is not so much that large amounts of money undermine the consent of *participants*. It is rather that if pharmaceutical firms and research labs are permitted to pay people enough money to participate in extremely risky experiments, and some of these experiments produce death and disease in a considerable number of participants, many people might lose trust in a profession that is supposed to cure disease rather than cause it.

We acknowledge this as a real challenge. But we think that a certain amount of skepticism toward medical authorities is desirable rather than objectionable. The trick for patients is to defer to the advice of experts when appropriate, but also to question their advice and think through the risks of enrolling in an experiment or in seeking treatment as a patient. This is part of the human condition. So the fact that some microbial challenge studies produce more harm than good should not give us reason to give up on testing new antibiotics or vaccines, or simply to accept that people will lose trust in companies that develop bad products. We should instead direct our efforts at teaching citizens how science works, and making the results of studies – as well as the decision procedures of IRBs to permit studies – as transparent as possible.

## 4 CONCLUSION

We are not making the argument that, if left alone, markets will solve all of our problems. In fact, we support significant investments by government in basic science research in order to encourage breakthroughs that pharmaceutical firms can translate into treatments. Instead, we are arguing that, along with a suite of policies that help conserve existing antibiotics and stimulate the development of new treatments,24Anomaly, J. (2017). Ethics, antibiotics, and public policy. *Georgetown Journal of Law and Public Policy, 15*, 999–1015. we should also allow researchers to use any amount of money they see fit to attract research participants. While we agree that some regulations might be desirable on who qualifies to participate, we think there are no moral limits on the amount of risk or money participants should be allowed to accept to take part in clinical trials.

Although our argument can be applied to most medical research, we think that it is especially compelling for treatments with the power to save and extend millions of lives. Infectious diseases have historically been responsible for more human suffering than almost any other force in nature. Tackling infectious diseases, including those resistant to antibiotics, will require extraordinary investments of resources, and may require us to change our attitudes about paying people to participate in research.

## CONFLICT OF INTEREST

The authors declare no conflict of interest.

